# Anemia That Presented with Desaturation: A Focus on Core Concepts

**DOI:** 10.1155/2021/5583840

**Published:** 2021-05-17

**Authors:** Lakshmi Shobhavat, Antonio D'Costa, Karthik Shroff

**Affiliations:** Bai Jerbai Wadia Hospital for Children, Parel, Mumbai, India

## Abstract

*Background*. Methemoglobinemia is a potentially life-threatening condition which presents with cyanosis and characteristic “chocolate-coloured blood.” Although a co-oximetry would give a prompt diagnosis, there have been multiple reports of misdiagnosing this treatable condition—from being diagnosed as sepsis to asthma and even being operated for “ruptured ectopic pregnancy.” Here, we report a case which presented without the classical signs of poisoning and methemoglobinemia—without vomiting, cyanosis, or chocolate-coloured blood. We also discuss the common misconceptions regarding anemia physiology and the pitfalls in diagnosing this condition and warn the reader regarding the reflexive use of antidotes like methylene blue. *Case Presentation.* A well-grown 3-year old boy presented with an acute history of irritability, cola-coloured urine, and desaturation on examination. The child was pale, with tachypnoea and in failure. Blood smear was suggestive of severe hemolytic anemia. Methemoglobinemia was diagnosed on co-oximetry. By focussing on physiologic principles of management rather than a specific antidote, the child was discharged home, well and active within 3 days of intensive care admission.

## 1. Introduction

Methemoglobinemia is a potentially life-threatening condition which presents with cyanosis and characteristic “chocolate-coloured blood.” Although a co-oximetry would give a prompt diagnosis, there have been multiple reports of misdiagnosing this treatable condition. There have been cases of this condition being treated as sepsis [1], being misdiagnosed as asthma for 5 years and treated with bronchodilators [2], and even being operated for “ruptured ectopic pregnancy” [3, 4]. Here, we report a case of methemoglobinemia presenting atypically, diagnosed on co-oximetry and managed with blood transfusion and high-dose ascorbic acid. We further go on to discuss and clarify the concepts behind this condition.

## 2. Case Presentation

A 3-year old boy presented to the emergency department with an acute history of 4-5 episodes of hematuria over the past 6 hours and a history of parents' noticing pallor since a day before. He was apparently well and healthy the previous day. The child was irritable on presentation, with tachycardia and tachypnoea. On primary assessment, he had warm extremities, a palpable liver, minimal crepts, and a gallop rhythm. The blood pressure was maintained. The presiding doctor noticed that the child's saturation on the pulse oximeter to be ∼68–72%, and the child was immediately put on non-rebreathable mask at 10 litres/minute. The saturation rose to ∼74–80%. There was no visible cyanosis over fair skin, and the sensorium was normal with a Glasgow Coma Scale (GCS) of 15/15.

The complete blood count revealed a haemoglobin of 3.8 g/dL, with a haematocrit of 13.1% and a normal bleeding profile. Total bilirubin was 3.7 mg% with an indirect component of 2.7 mg%. While the pulse oximeter continued to show a poor saturation (∼80%), the blood gas reports painted an entirely different picture: pH, 7.5; pCO_2_, 24.9 mmHg; pO_2_, 215.3 mmHg; HCO_3_, 19.2 mmol/L; lactates, 3.5 mmol/L; and saturation (SaO_2_), 99.8%. With a wide saturation gap, abnormal forms of haemoglobin were suspected, and a co-oximetry on arterial blood was requested—which came positive for methemoglobinemia (MetHb: 10.4%), with an O_2_Hb (oxygenated Hb) of 87.4% and an HHb (deoxygenated Hb) of 0.3% (Table 1).

The peripheral smear was suggestive of acute haemolysis (with iron deficiency anemia): severe anisocytosis, polychromasia, spherocytes, severe hypochromia, and nucleated red blood cells (RBC).

Glucose-6 phosphate dehydrogenase (G6PD) was normal.

In view of cardiac failure, injection furosemide at a dose of 1 mg/kg was administered. The child was transfused packed red blood cells at 5 cc/kg, and ascorbic acid at a dose of 10 mg was started. We did not administer methylene blue, as the threshold percentage of methemoglobin (MetHb) was not high enough to warrant, and any risk of haemolysis due to an oxidising agent would further jeopardise the child.

The child improved over the course of the next 2 days, requiring two more blood transfusions and was discharged on the 4th day after admission.

We could not find any concrete source of methemoglobinemia poisoning for the child: as the child came from an urban slum neighbourhood, we suspected contamination of their water source and tested the water samples for nitrates [5] and nitrites, which were NIL.

**Figure 1:**
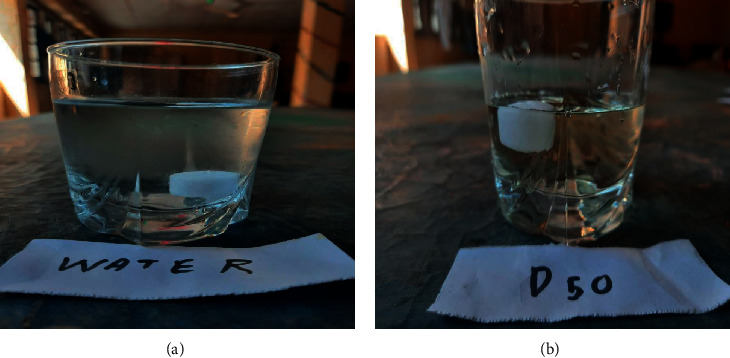
While camphor floats in water, both paradichlorobenzene and naphthalene will sink. In 50% dextrose, both naphthalene and camphor will float, while paradichlorobenzene will sink.

A history of mothball use in the household was elicited. With a mild presentation and no history of vomiting, diarrhoea, and seizure, we suspected paradichlorobenzene (PDB) mothball poisoning [6] over naphthalene and camphor. We performed a point-of-care test to differentiate between the three. As per work by Moss et al. [7] (Table 2), camphor, PDB, and naphthalene can be differentiated based on their density using a combination of water and 50% dextrose. Since the mothball sank in water, but floated in the D50 solution, it was concluded as consisting of naphthalene (Figure 1).

## 3. Discussion

Cases of haemoglobinopathies need not always present with frank cyanosis, nor all cases of methemoglobinemia be treated with methylene blue.

Although these patients present with desaturations and refractory hypoxemia, reflexive intubations should be restrained, and the clinician should be aware of this diagnosis. We would now like to discuss our reasoning and logic towards the management of this child from him presenting in failure to a satisfactory day-5 discharge. A few concepts need to be discussed first.

### 3.1. How Did the Cyanosis Evade Detection?

Although a more common feature in G6PD-deficient individuals (due to strong oxidising toxins such as naphthalene inducing direct oxidative stress on RBC membranes [8]), there have been a few reported cases of hemolytic anemia in patients with normal G6PD levels [9].

Our child showed haemolysis on his peripheral smear, which had dropped his haemoglobin levels down to 3.8 gm/dL (possibly in an already nutritionally deficient, anemic child).

While cyanosis is one of the most classic findings in methemoglobinemia, it is visible only when deoxyhemoglobin levels are 5.0 gm/dl or higher [10] (recent evidence suggests levels of 2.0 gm/dl may reliably produce cyanosis [11]) or methemoglobin levels are above 1.5 g/dL (approximately equal to 10–15% methemoglobin in a patient with normal haemoglobin levels) [12].

In our case with an Hb of 3.8 gm/dL, of which 10.4% was MetHb (0.4 gm/dL) and 0.3% was HHb (0.01 gm/dL), the methemoglobin and deoxyhemoglobin would not have been high enough to produce the characteristic bluish hue.

### 3.2. Rationale for Management of Failure

**Figure 2:**
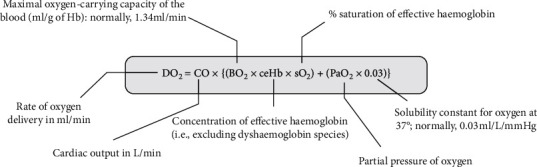
Rate of oxygen delivery and its relation to cardiac output and oxygen content of blood.

Referencing the above equation (Figure 2) [13] with a target to improve oxygen delivery to hypoxic tissues, as cardiac output (stroke volume × heart rate) has already been optimised by the body through an increased heart rate, our limiting factor in severe anemia becomes the concentration of haemoglobin per unit of blood.

Haemoglobin is the molecule which actually binds and transports oxygen in the blood.

The wisest then, in an anemic child, would be to focus on the “ceHb” (concentration of effective haemoglobin) and “sO2” (% saturation of effective haemoglobin) part of this equation through adequate and slow blood transfusions, working to raise “effective” haemoglobin (and also as a consequence leading to a decrease in the percentage of MethHb).

### 3.3. Clearing the Concept of Oxygen Saturation

We would also like to point out that anemia, per se, cannot cause arterial desaturation.

The concept of saturation applies to the oxygen-haemoglobin dissociation curve.

**Figure 3:**
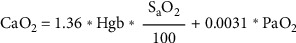
Oxygen content.

Let us reference the 2nd portion of the previous formula (Figure 2):

The above (Figure 3) is the formula for oxygen content in blood.

Oxygen is present in blood in two forms: bound to haemoglobin and the free oxygen molecules dissolved in blood. With these two systems, the blood can transport approximately 20 ml of oxygen for every 100 ml of blood, to provide for tissue requirements. The free oxygen gas molecules (exerting a partial pressure-Po2) and the haemoglobin bound form are related to each other through the oxygen haemoglobin dissociation curve (ODC), the latter represented as oxygen saturation.

As the haemoglobin molecule is limited to 4 sites for binding to oxygen, this bound form is represented more intuitively as saturation—the amount of sites filled by oxygen and represents how “saturated” the haemoglobin is—i.e., when all 4 sites are bound, we would have 100% saturation for a haemoglobin molecule. Oxygen saturation is a percentage of oxyhemoglobin (O_2_Hb) to total Hb [14].

**Figure 4:**
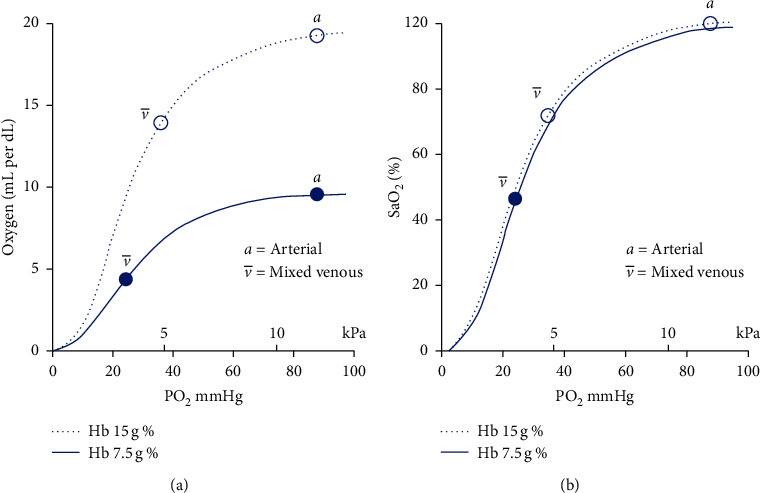
Relation of anemia to oxygen content and oxygen-haemoglobin dissociation curve (ODC).

The core concept here is that, in anemia, we have a decrease in haemoglobin and hence a reduction in the total haemoglobin binding sites for oxygen. Here arises the misconception that saturation should hence fall. But these binding sites still retain their affinity for oxygen and “saturate” themselves as previously. As saturation is merely a ratio of oxygen bound to haemoglobin (the binding sites may be decreased, but they are still “saturated” the same), anemia cannot affect arterial saturation (Figure 4) [15], per se.

Anemia, though, affects the oxygen delivery to tissues by decreasing total oxygen content (ml/dL) (Figure 3) and can cause hypoxia (reduced level of tissue oxygenation). Indeed, in anemia, there is a fall in venous saturation (Figure 4). As the oxygen reserve decreases due to low haemoglobin, tissue extraction leads to a fall in the venous pO_2_. As per the ODC, the fall in pO_2_ leads to less oxygen molecules binding haemoglobin and hence it is lower saturation at the venous end (Figure 4) [15].

Furthermore, we would like to briefly discuss the concept behind the discrepancies in saturation readings between the pulse oximeter and co-oximetry observed.

A pulse oximeter is a simple device that has two LEDs: a red (660 nm) and an infrared (930 nm).

These wavelengths are emitted and pass through tissue and get absorbed by the blood perfusing them.

As the light passes through, oxygenated haemoglobin absorbs more infrared light and allows more red light to pass through, while deoxygenated haemoglobin allows more infrared light to pass through and absorbs more red light [16].

**Figure 5:**
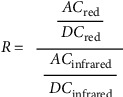
‘*R*' ratio. AC: pulsatile arterial blood absorbance; DC: tissue + capillary blood + venous blood + nonpulsatile arterial blood absorbance.

A microcontroller then calculates the ratio (Figure 5) accounting for tissue, capillary, and venous blood absorbance.

This ratio (*R*) (Figure 5) is compared to an internally stored lookup table to output a corresponding saturation read out:

- SaO2 100% = *R* 0.4
- SaO2 85% = *R* 1
- SaO2 0% = *R* 3.4

A curious case occurs in methemoglobinemia. The ferric state of haemoglobin has a very high absorbance to both red and infrared light, virtually always resulting in an *R* of 1.0 in the pulse oximeter. This leads to the pulse oximeter underestimating the oxygen saturation of the blood or the read out hovering around 80% (approaching and plateauing at 85% with methemoglobin levels of 30% or higher) [17].

Blood gas analysers, on the other hand, measure arterial saturation (SaO2) and have been designed to assume that all haemoglobin is either oxyhemoglobin or deoxyhemoglobin and hence overestimate blood saturation in a patient with methemoglobinemia.

**Figure 6:**
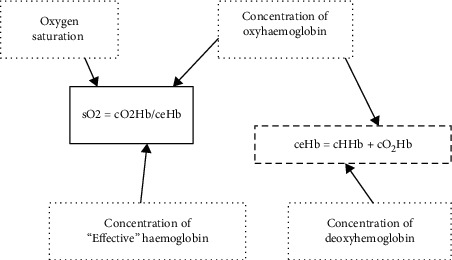
Oxygen saturation. ceHb (effective haemoglobin) excludes the fractions of carboxy‐ and methemoglobin, which a blood gas analyzer cannot quantify accurately [18].

These devices use this oversimplified equation (Figure 6):

This difference between the SpO_2_ reading and the SaO_2_ read out gives us what is called the “saturation gap.” A saturation gap of >5% should point towards haemoglobinopathy, and a co-oximetry should be ordered for [19].

### 3.4. Methemoglobinemia Does Not Always Warrant Methylene Blue

While methylene blue remains the mainstay of treatment for methemoglobinemia, we would like to point out that it should be used cautiously. Present work suggests a cutoff of 20% in symptomatic and 30% in asymptomatic patients [20].

As we have demonstrated in the treatment of this case, alternative approaches such as prompt blood transfusions aid in decreasing the percentage of methemoglobin and improving the clinical status of the patient, without the need for methylene blue administration. Although very minimal supportive evidence exists for the use of vitamin C in high doses [21], ascorbic acid being a strong reducing agent could theoretically help in reducing methemoglobin levels.

One should also note that methylene blue itself is an oxidising agent and could lead to further RBC membrane damage and extravascular haemolysis worsening the anemia in a haemoglobin-depleted patient.

## 4. Conclusion

The purpose of this case report is two fold: first, it is to warn the physician that not all cases of methemoglobinemia could present with cyanosis, being masked by the haemolysis-induced anemia. Clinical suspicion should always be aroused in cases presenting with refractory hypoxemia.

Secondly, methylene blue is not always the “go to” remedy. It is best to follow physiologic principles of management and consider alternative therapies for improved outcome at least risk to the patient.

## Figures and Tables

**Table 1:** Lab investigations.

Date	22/01/2021 (on admission)	23/01/2021
Haemoglobin (g/dL)	3.8	6.1
Hematocrit (%)	13.1	18.9
pH	7.5	7.5
pCO_2_ (mmHg)	24.9	31.4
pO_2_ (mmHg)	215.3	89.8
HCO_3_ (mmol/L)	19.2	24.3
Lactate (mmol/L)	3.5	—
SaO_2_ (%)	99.8	97.8
MetHb (%)	10.4	—
O_2_Hb (%)	87.4	—
HHb (%)	0.3	—

**Table 2:** Point-of-care test to differentiate between camphor, PDB, and naphthalene.

	Water	50% dextrose
Camphor	Floats	Floats
PDB	Sink	Sink
Naphthalene	Sink	Float

## Data Availability

In view of the hospital policy on patient privacy, the data can be shared only on request and on a per-case basis.
